# Supplementary material for: MetaRibo-Seq measures translation in microbiomes
Source: Nat Commun. 2020 Jun 29;11:3268. doi: 10.1038/s41467-020-17081-z (PMC7324362; doi:10.1038/s41467-020-17081-z)
Supplement: Supplementary file 10 — Supplementary Data 7 [file 41467_2020_17081_MOESM10_ESM.zip › File2/Confidence_High_Taxonomy/191628_out.krona.html]

Javascript must be enabled to view this page.

members
magnitude
magnitudeUnassigned
count
unassigned
taxon
rank

191628\_out

3

2
3
superkingdom

1239
2
phylum

class
186801
2

order
2
186802

family
31979
1

1
1485
genus


SRS015663\_contig\_number\_14440
species
2293052
1

family
1
186803

genus
265975
1

237576
1

SRS078182\_contig\_number\_50830
species

201174
1
phylum

84998
1
class

order
1
84999

family
84107
1


SRS098073\_contig\_number\_contig-100\_21789.21790
genus
102106
1
